# Protective effect of Saikosaponin D modulating endoplasmic reticulum stress mediated by TLR4/MyD88/NF-κB/NLRP3 pathway on cholestatic liver injury

**DOI:** 10.1186/s41065-026-00669-8

**Published:** 2026-04-11

**Authors:** XianMeng Meng, Wei Chen

**Affiliations:** 1 Liaoning University of Traditional Chinese Medicine, No.79 Chongshan East Road, Huanggu District, Shenyang City, Liaoning Province 110847 China; 2 Affiliated Hospital of Liaoning University of Traditional Chinese Medicine, No. 33 Beiling Street, Huanggu District, Shenyang City, Liaoning Province, 110032, China

**Keywords:** Saikosaponin D, Endoplasmic reticulum stress, Cholestatic liver injury, Inflammatory signaling

## Abstract

**Objective:**

Cholestatic liver injury (CLI) has lacked a potent treatment due to its incompletely understood pathogenesis. Saikosaponin D (SSD), the main active ingredient of the traditional Chinese medicine Bupleuri Radix, has significant anti-inflammatory and hepatoprotective effects and inhibits hepatic fibrosis. This study specifically investigated the protective effects of SSD in CLI and its related mechanisms.

**Methods:**

In bile duct ligation (BDL)-induced CLI in mice, the protective effect of SSD was assessed by serum biochemical index analysis, HE staining of liver tissue, Sirius red staining, immunohistochemistry and ELISA. Bile acid-induced human HepG2 cell line was taken to study the protective effect and potential mechanism of SSD against CLI in vitro. TLR4/MyD88/NF-κB/NLRP3 pathway-related proteins in mouse liver tissues or HepG2 cells were detected by Western blot or immunofluorescence.

**Results:**

SSD dose‑dependently improved liver function and reduced hepatocyte necrosis, collagen deposition, and apoptosis. It attenuated oxidative stress injury, inflammatory responses, and endoplasmic reticulum stress (ERS) in BDL mice. SSD also mitigated bile acid‑induced injury and ERS in HepG2 cells. Moreover, SSD suppressed the activation of the TLR4/MyD88/NF‑κB/NLRP3 pathway. The TLR4 inhibitor TAK‑242, similar to SSD, ameliorated bile acid‑induced HepG2 cell injury and ERS, whereas the TLR4 agonist LPS counteracted the protective effects of SSD and its inhibition of ERS in bile acid‑stimulated HepG2 cells.

**Conclusion:**

SSD attenuates CLI, an effect that may be associated with the suppression of the TLR4/MyD88/NF‑κB/NLRP3 pathway and the subsequent inhibition of ERS.

**Supplementary Information:**

The online version contains supplementary material available at 10.1186/s41065-026-00669-8.

## Introduction

Bile is produced by the liver and excreted into the intestine via the biliary system, where it plays an important role in promoting nutrient absorption and toxic excretion. However, bile metabolism disorders can cause large accumulation of bile components in the liver and blood, resulting in cholestatic liver injury (CLI) [1–3]. CLI can be divided into intrahepatic and extrahepatic types. Among them, extrahepatic CLI is mainly caused by biliary malformations, stones or tumors compressing the bile ducts, etc. Intrahepatic CLI has a complex etiology, which can be caused by viral infections, metabolism, genetics, and immunity. Surgical interventions can improve the condition of most patients with CLI due to biliary malformations, stones, or tumors compressing the bile ducts. For patients suffering from CLI of unknown pathogenesis, such as primary biliary cholangitis or primary sclerosing cholangitis, despite some clinical symptom improvements with ursodeoxycholic acid and obeticholic acid, some CLI patients still experience progression to liver fibrosis, cirrhosis, and even liver failure [4, 5]. Therefore, it is urgent to investigate the common pathogenesis of CLI caused by various etiologic factors and to find effective targets for intervention.

The endoplasmic reticulum is an important organelle responsible for protein synthesis, folding, assembly and secretion [6]. The aggregation of misfolded or unfolded proteins in the endoplasmic reticulum leads to a stress response in the endoplasmic reticulum called endoplasmic reticulum stress (ERS) [7]. The endoplasmic reticulum initiates a protective cellular mechanism known as the unfolded protein response to reestablish its homeostasis in reaction to ERS (UPR). However, if the stress is more severe or prolonged, the cell death pathway is activated [8]. Hepatocytes are rich in both smooth and rough endoplasmic reticulum and are highly vulnerable to ERS. Research indicates that ERS and the UPR signaling pathway are key factors in CLI and hold significant potential for therapy [9–11].

Bupleuri Radix is a traditional Chinese medicine widely used in China and other Asian countries for the treatment of chronic liver inflammation and viral hepatitis. Recently, more than 100 Saikosaponins have been extracted from Chaihu, with Saikosaponin A (SSA) and SSD identified as the main bioactive compounds. SSD is a triterpenoid saponin with multiple pharmacological effects, including anti-inflammatory, antioxidant, anti-apoptotic, anti-fibrotic, and liver-protective properties [12]. Studies have demonstrated the strong therapeutic potential of SSD for liver diseases including non-alcoholic fatty liver disease, alcoholic liver disease, drug-induced liver injury, and liver fibrosis [12–14]. However, no study has elucidated the protective role and potential mechanisms of SSD in CLI.

Toll-like receptors (TLRs) are pattern recognition receptors crucial for triggering inflammation and developing immunity. TLR4, an important member of the TLRs family, enters downstream signaling through the transmembrane structure via the carboxyl terminus of MyD88, and ultimately activates NF-κB to promote the release of inflammatory factors, which is a key link in liver inflammatory diseases [15]. This series of reactions induces hepatocyte apoptosis or necrosis, which ultimately leads to irreversible liver injury. TLR4 pathway is involved in CLI, and inhibition of TLR4 effectively attenuates bile duct ligation (BDL)-induced liver injury [16]. The modulation of the TLR4/NF-κB pathway by SSD may effectively ease depressive-like behaviors related to inflammation triggered by LPS [17]. Further, SSB2 shows anti-neuroinflammatory properties via the TLR4/NF-κB pathway, relying on GPX4 [18], and SSA interferes with the TLR4/MyD88/NF-κB signaling by regulating dopaminergic neurodegeneration resulting from microglia activation [19].

Therefore, the present study aimed to elucidate the therapeutic effects of SSD on BDL-induced CLI in vivo and bile acids-treated HepG2 cells in vitro and to further explore its therapeutic effects in relation to the TLR4-related pathway.

## Materials and methods

### Animal model and drug administration

Approximately 8-week-old male C57BL/6J mice, with a weight range of 20–25 g, were acquired from Shanghai Jiesijie Laboratory Animal Co. Ltd. (Shanghai, China). For one week, the mice were acclimated and housed in an SPF setting with temperatures between 21 and 25℃, a 12-hour day/night cycle, and free access to food and water. This experiment was approved and reviewed by Animal Ethics Committee of Liaoning University of Traditional Chinese Medicine.

According to the method detailed in [20] for BDL, mice received an intraperitoneal injection of 1% pentobarbital sodium at 40 mg/kg to induce anesthesia. The surgical area on the abdomen was prepared with a skin-preparation knife, and the tissue was cut along the midline of the mouse abdomen layer by layer until the liver was fully exposed. A wire was threaded through the sternum and lifted upward and secured, and a chest expander was placed into the abdominal cavity to expose the surgical field. Using a sterile cotton swab, the common bile duct was exposed using a sterile cotton swab and detached using forceps. Two 4 − 0 surgical sutures were placed under the common bile duct and knotted and cut separately. The abdominal cavity was cleared and the intra-abdominal organs were reset. The abdomen was closed using suture staples and the wound was disinfected. After surgery, the mice were resuscitated in a clean, aerobic, constant temperature environment. In the sham-operated group, the common bile duct was left unligated after the abdomen was opened, with all other procedures remaining identical.

Mice were randomly divided into 5 groups of 10 mice each: Sham group, CLI group, and SSD low, medium, and high dose treatment groups. The sham group experienced sham ligation, while the CLI group had CLI induced through BDL, and the SSD treatment groups received intraperitoneal injections of SSD at low, medium, and high doses of 1, 1.5, and 2 mg/kg, respectively (Sigma-Aldrich, MO, USA), after BDL surgery. The doses of SSD were determined through a preliminary dose-response study that assessed various concentrations within a safe and physiologically appropriate range. The chosen doses elicited a therapeutic response and were well-tolerated, with no detectable detrimental effects. Furthermore, the dosing regimen is supported by its alignment with well-established SSD administration protocols in murine models as previously reported [13, 14, 21]. The Sham and CLI groups were given the same intraperitoneal saline dose for 7 consecutive days. At the end of the experiment, the mice were euthanized, and the serum and liver were removed for subsequent experiments.

### Serum biochemical analysis

Serum alkaline phosphatase (ALP), alanine aminotransferase (ALT), total bilirubin (TBIL), azelaic transaminase (AST), and total bile acid (TBA) were determined using commercially available kits (Nanjing Jianjian Bioengineering Institute, Nanjing, China).

### HE staining

Paraffin sections of liver tissue were routinely dehydrated by gradient alcohol staining, cleared in xylene, stained with hematoxylin for 3–5 min, differentiated by 1% hydrochloric acid in alcohol for 20 s, and immersed in 1% ammonia for 30 s. The samples were restained in l% eosin solution for 5 min, routinely dehydrated, cleared, dried, and sealed. The structure and morphology of liver tissue were viewed through a light microscope (Olympus, Japan), and the necrotic area was quantified by ImageJ software (National Institutes of Health, USA).

### TUNEL staining

Liver tissue paraffin sections were routinely deparaffinized, digested with proteinase K, and stained using Fluorescent TUNEL Apoptosis Detection Kit (Beyotime, Shanghai, China). The nuclei of the cells were restained with DAPI and observed under a fluorescence microscope (Olympus). The number of TUNEL-positive cells (apoptotic cells) was counted by Image J software.

### Sirius red staining

Paraffin sections of liver tissue were routinely deparaffinized and incubated with drops of Sirius red staining solution (Yeasen, Shanghai, China) for 1 h at room temperature. They were stained with hematoxylin for 30 s, rinsed with running water for 10 min, dehydrated with ethanol gradient, cleared with xylene, and sealed to observe fibrosis under an optical microscope. The area of Sirius red staining was quantitatively analyzed by ImageJ software.

### Immunohistochemistry (IHC)

Liver tissue sections were routinely deparaffinized. Endogenous peroxidase was eliminated by H_2_O_2_, and antigen was retrieved by microwave. Then, the sections were blocked with 10% normal goat serum for 30 min, incubated with primary antibody GRP78/BIP (1:200, 11587-1-AP, Proteintech) for 60 min, and added with secondary antibody (Proteintech) for 30 min. Afterward, the sections were stained with hematoxylin and sealed with neutral resin after DAB color development. The samples were visualized under a light microscope and photographed.

### Immunofluorescence staining

Liver tissue sections were deparaffinized, heat twice, and blocked with normal serum. Primary antibodies against TLR4 (1:400, 19811-1-AP, Proteintech), MyD88 (1:300, ab2064, Abcam, MA, USA), p-NF-κB p65 (1:800, #3033S, CST) and NLRP3 (1:300, 330109-1-AP, Proteintech) were incubated overnight at 4 °C protected from light. Sections were then incubated with the appropriate fluorescence-conjugated secondary antibody (1:500, Invitrogen, USA) for 2 h, restained with DAPI for 15 min, and viewed under an inverted Olympus FV1200 confocal microscope system. Fluorescence intensity was measured using ImageJ software.

### MDA, SOD and GSH assays

Placed in cold saline at a 1:10 weight-to-volume ratio, the livers were homogenized and centrifuged at 3500 r/min for 10 min. MDA, SOD and GSH in liver tissue homogenates were measured using kits (Solarbio, Beijing, China).

### Determination of inflammatory factors

TNF-α, IL-1β and IL-6 in liver tissue homogenates were detected using the ELISA kits (Nanjing Jianjian Bioengineering Institute).

### Cell culture and treatment

The human hepatoma cell line HepG2 (ATCC, VA, USA) were cultured in DEME (Thermo Fisher Scientific, MA, USA) containing 10% fetal bovine serum (Thermo Fisher Scientific), 1% penicillin (10,000 U/L)/streptomycin (10,000 µg /ml) (Thermo Fisher Scientific). HepG2 cells were exposed to 200 µM chenodeoxycholic acid (CDCA, Sigma-Aldrich) for 12 h to simulate CLI-like damage, as previously outlined [22]. To investigate the protective effect of SSD against CDCA-induced injury, HepG2 cells were treated with 5 µM SSD and 200 µM CDCA for 12 h.

To investigate the role of ERS in SSD against CLI, HepG2 cells were incubated with the ERS agonist tunicamycin (TM, 20 µg/mL, Sigma) or inhibitor Tauroursodeoxycholic Acid (TUDCA, 50 µM, Sigma) for 30 min, followed by 12 h of incubation with 5 µM SSD and 200 µM CDCA. To assess the role of the TLR4/MyD88/NF-κB/NLRP3 pathway in SSD’s defense against CLI, the TLR4 inhibitor TAK-242 (1 µM, Sigma) or TLR4 agonist lipopolysaccharide (LPS, 100 ng/mL, Sigma) was incubated for 30 min, followed by a 12-hour incubation with 5 µM SSD and 200 µM CDCA.

### CCK-8

Cell viability was assessed using the CCK-8 kit (Solarbio). Cells were grown at 2 × 10^4^ cells/well in 96-well plates overnight. After corresponding treatment, 10 µL of CCK-8 solution was added directly into 100 µL of DMEM and incubated for 1–2 h. Absorbance was measured at 450 nm.

### LDH assay

Cytotoxicity was detected using LDH release assay kit (Beyotime). The supernatant of cells (120 µL) was mixed with LDH detection solution (60 µL). After incubation for 30 min, the absorbance was measured at 490 nm.

### Apoptosis detection

Cells were added to 500 µL of pre-cooled 1 × binding buffer, 5 µL of Annexin-V-FITC, and 2.5 µL of PI (Annexin V/PIApoptosis Analysis Kit, Beyotime). After incubation for 15 min away from light, cells were assayed for apoptosis rate on a flow cytometer (BD FACSArial I cell sorter, BectonDickinson, NJ, USA).

### Western blot

Proteins were extracted by RIPA lysis buffer (Beyotime) and quantified by BCA method. A 2:1 mixture of extracted sample proteins and loading buffer was boiled, denatured, and then processed using sodium dodecyl sulfate-polyacrylamide gel electrophoresis. The proteins were transferred to polyvinylidene fluoride membranes, blocked, and incubated at 4 °C with primary antibodies ATF4 (1:1000, 10835-1-AP, Proteintech), CHOP (1:1000, #2895, CST), GRP78 (1:1000, 11587-1-AP, Proteintech), TLR4 (1:1000, 19811-1-AP, Proteintech), MyD88 (1:1000, ab2064, Abcam), p-NF-κB p65 (1:1000, #3033S, CST), NF-κB p65 (1:1000, #8242, CST), NLRP3 (1:1000, ab263899, Abcam), and β-actin (1 µg/mL, ab8226, Abcam) overnight. Then the corresponding horseradish peroxidase-labeled secondary antibody (1: 5000, Abcam) was incubated at room temperature for 1 h. Protein bands were exposed and developed by ECL chemistry, and target proteins was calculated using ImageJ software.

### Statistical analysis

All data were processed using GraphPad Prism 9 (GraphPad Software, CA, USA), shown as mean ± standard deviation. Pearson analysis was conducted for correlation, with a t-test used for two-group comparisons and one-way ANOVA for multiple group comparisons. Two-by-two comparisons after ANOVA were performed using Tukey’s multiple comparisons test. *P* < 0.01 indicated that the differences were statistically significant.

## Results

### SSD ameliorates BDL-induced CLI

**Fig. 1:**
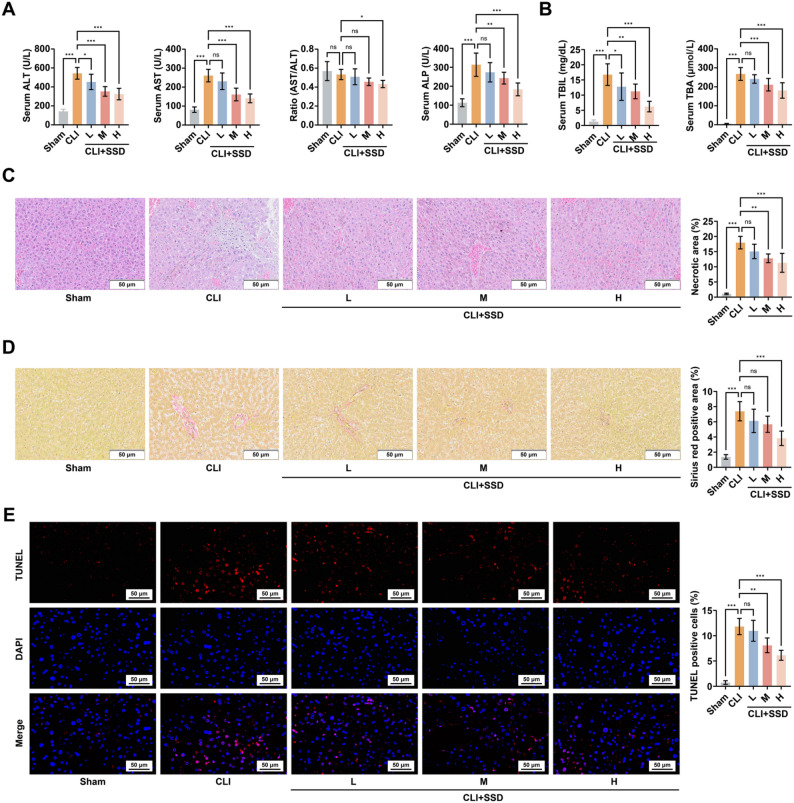
SSD ameliorates BDL-induced liver injury. **A**: Commercial kits were used to measure serum levels of AST, ALT, ALP, and the AST/ALT ratio in CLI mice following SSD treatment (*n* = 10); **B**: Commercial kits were used to detect serum levels of TBIL and TBA in CLI mice following SSD treatment (*n* = 10); **C**: Representative images of HE staining and quantitative analysis of necrotic area of liver tissues of mice (*n* = 5); **D**: Representative images of Sirius red staining and quantitative analysis of stained area of liver tissues of mice (*n* = 5); **E**: Representative images of TUNEL staining and quantitative analysis of the proportion of TUNEL-positive cells in liver tissues of mice (*n* = 5). Data are presented as mean ± SD. * *P* < 0.05, ** *P* < 0.01, *** *P* < 0.001, ns, not significant

Mice with BDL-induced CLI were administered different doses of SSD. Serum analysis indicated significant elevations in AST, ALT, ALP, TBIL, and TBA levels in the BDL model group. Following SSD treatment, all of these parameters exhibited varying degrees of improvement, accompanied by a reduction in the AST/ALT ratio (Fig. 1A, B). Compared to the sham group, HE staining indicated a significant expansion of necrotic areas in the liver tissue of CLI mice. Sirius red staining showed that BDL significantly increased liver fibrosis. Meanwhile, TUNEL staining analysis showed a significant increase in apoptotic cell death in the liver tissues of CLI mice. After SSD intervention, these histological changes were improved (Fig. 1C-E).

### SSD alleviates BDL-induced oxidative stress and inflammation

**Fig. 2:**
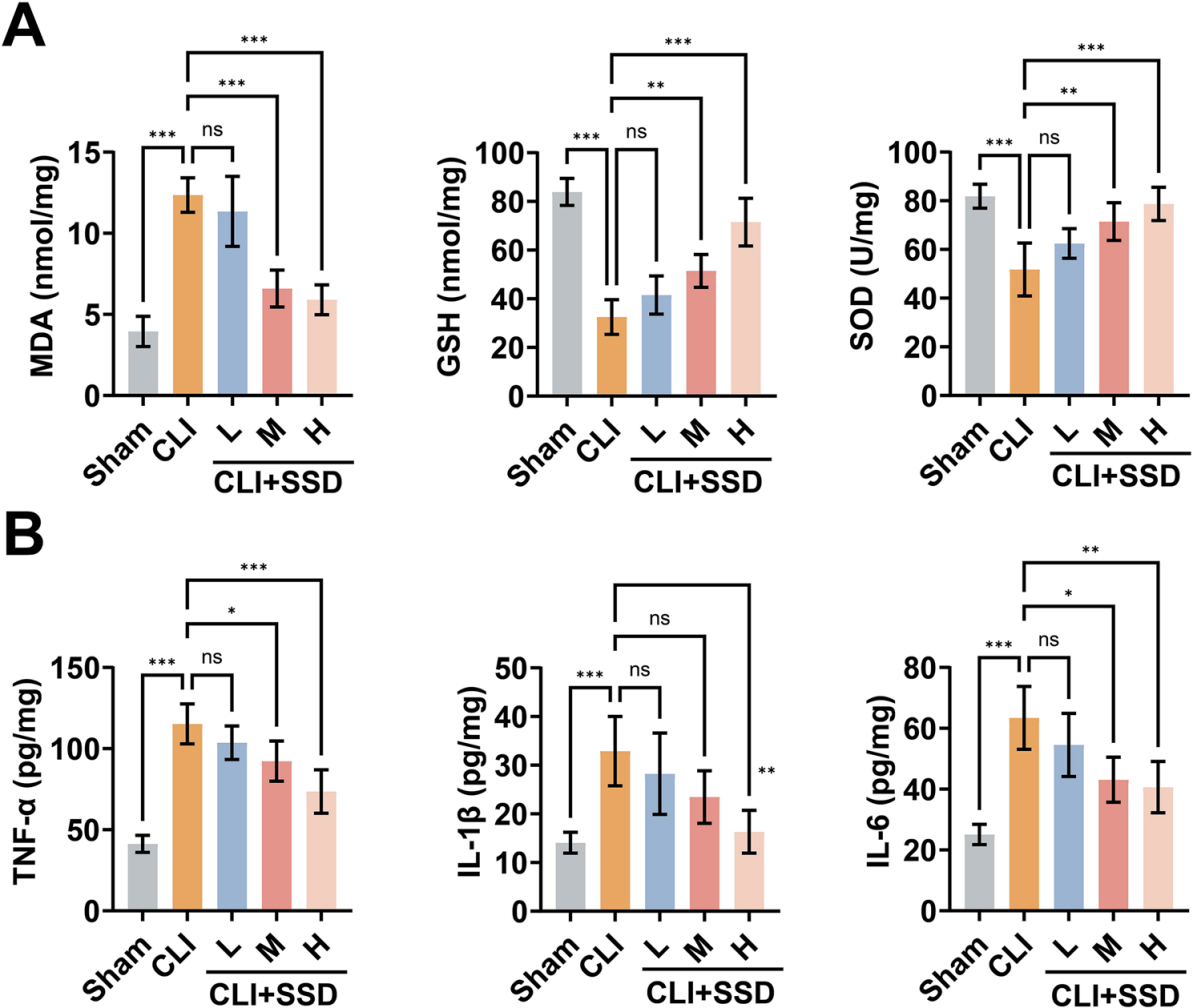
SSD alleviates BDL-induced oxidative stress and inflammation. **A**: Commercial kits to detect the effect of SSD on the levels of MDA, GSH and SOD in the liver tissues of CLI mice; **B**: ELISA to detect the effect of SSD on TNF-α, IL-1β, and IL-6 in the liver tissues of CLI mice. Data are presented as mean ± SD (n = 5).* *P* < 0.05, ** *P* < 0.01, *** *P* < 0.001, ns, not significant

Accumulation of bile acids in the liver induces oxidative stress, inflammation, hepatic stellate cell activation, and hepatocyte death [23]. Detection of oxidative stress-related indices in mouse liver tissues revealed (Fig. 2A) that MDA, a lipid peroxidation product, was significantly elevated in the liver tissues of CLI mice, while GSH and SOD were reduced. SSD intervention increased the intrahepatic levels of GSH and SOD, while decreasing MDA. ELISA for inflammatory mediators in liver tissues revealed (Fig. 2B) that BDL significantly induced the elevation of TNF-α, IL-1β, and IL-6, which were significantly inhibited by SSD.

### SSD inhibits BDL-induced ERS

**Fig. 3:**
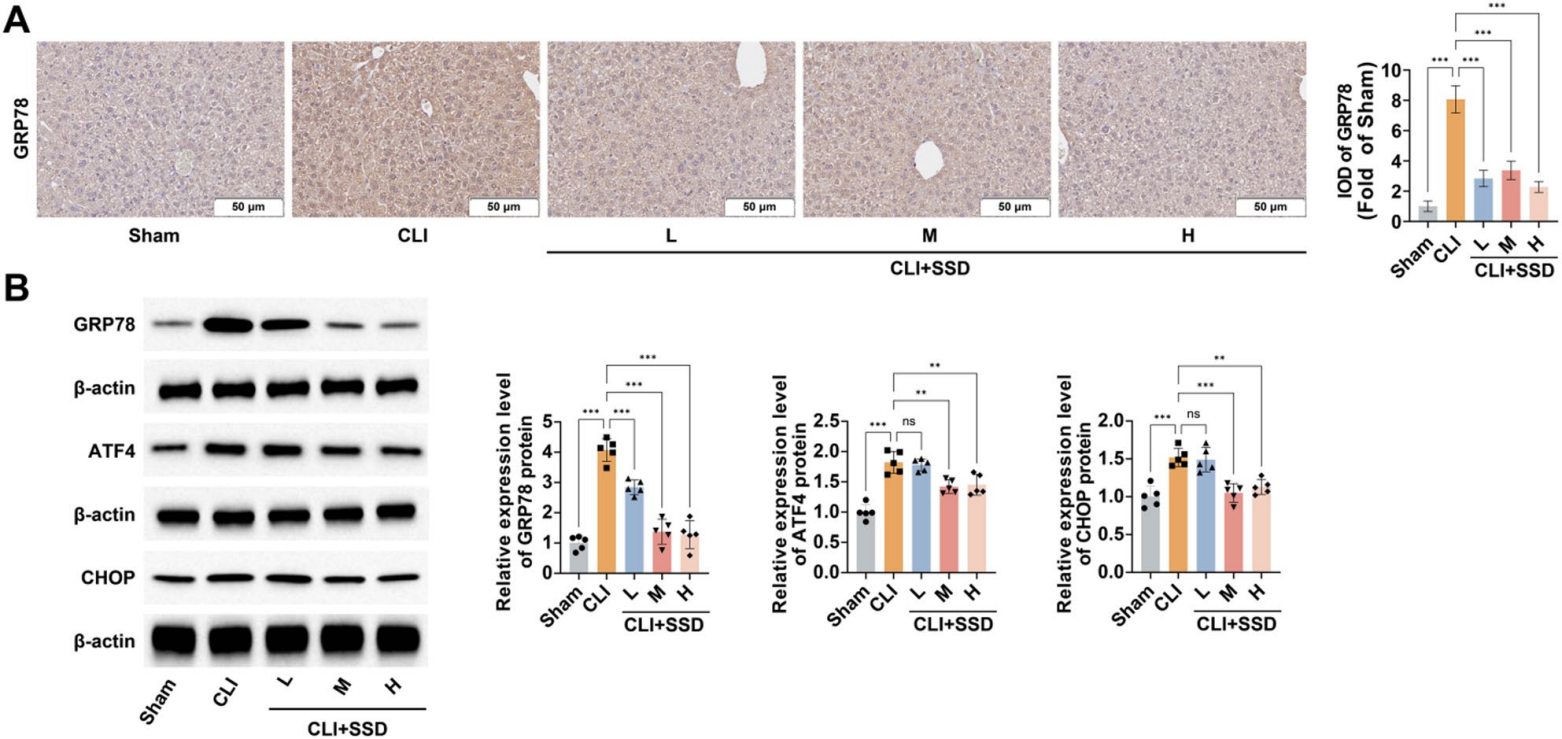
SSD inhibits BDL-induced ERS. **A**: Immunohistochemical detection of GRP78 expression in the liver tissues of CLI mice following SSD treatment, and the corresponding Integrated Optical Density (IOD) quantitative analysis; **B**: Western blot to detect the effect of SSD on the protein expression of ERS markers GRP78, ATF4 and CHOP in the liver tissues of CLI mice. Data are presented as mean ± SD (n = 5). * *P* < 0.05, ** *P* < 0.01, *** *P* < 0.001, ns, not significant

Excess bile acids in cholestasis lead to ERS, contributing to experimental CLI development [11]. Immunohistochemical analysis of the ERS marker GRP78 showed that GRP78 expression was significantly stronger in BDL livers than in sham control livers. It was evident that mice exhibited markedly increased ERS in response to CLI (Fig. 3A). In the liver tissues of BDL-treated mice, immunohistochemical analysis showed a significant increase in GRP78 expression (Fig. 3B). In contrast, SSD intervention inhibited BDL-induced ERS in the liver, with the extent of suppression depending on the dose (Fig. 3A, B).

### SSD inhibits bile acid-induced HepG2 cell injury and ERS

**Fig. 4:**
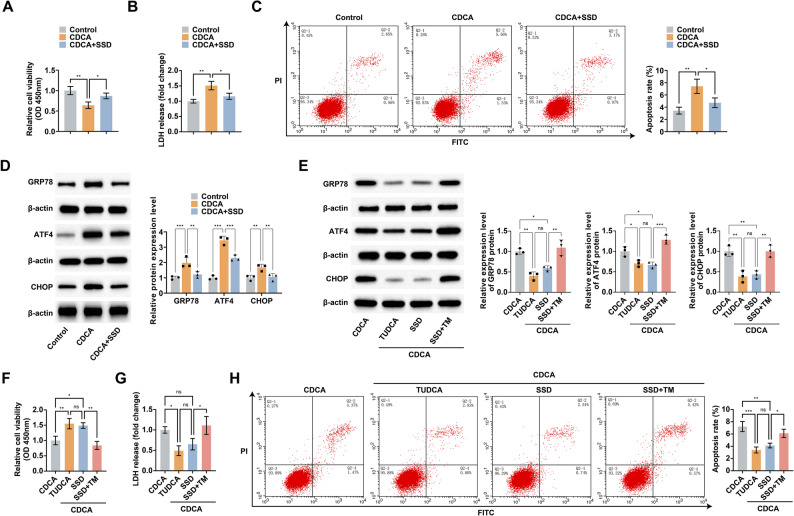
SSD inhibits bile acid-induced HepG2 cell injury and ERS. **A**: CCK8 to detect the effect of SSD on CDCA-stimulated HepG2 cell viability; **B**: LDH release to assess the effect of SSD on CDCA-stimulated HepG2 cell toxicity; **C**: Flow cytometry to detect the effect of SSD on the apoptosis rate of CDCA-stimulated HepG2 cells; **D**: Western blot to detect the effect of SSD on CDCA-stimulated HepG2 cell ATF4, CHOP and GRP78 protein expression; **E**: Western blot to detect the effect of TM or TUDCA on ATF4, CHOP and GRP78 protein expression in CDCA-stimulated HepG2 cells; **F**: CCK8 to detect the effect of TM or TUDCA on the viability of CDCA-stimulated HepG2 cells; **G**: LDH release to assess the effect of TM or TUDCA on CDCA-stimulated HepG2 cell toxicity; **H**: Flow cytometry to detect the effect of TM or TUDCA on the apoptosis rate of CDCA-stimulated HepG2 cells. Data are presented as mean ± SD (n = 3). * *P* < 0.05, ** *P* < 0.01, *** *P* < 0.001, ns, not significant

HepG2 cells were further stimulated with CDCA, a major component of bile acids, to mimic CLI in vitro to investigate the protective effects and potential mechanisms of SSD against CLI. CCK8 and LDH assays showed that SSD reversed CDCA-induced decrease in cell viability and elevated cytotoxicity (Fig. 4A, B). Flow cytometry results showed that SSD reduced the CDCA-induced elevated apoptosis rate (Fig. 4C). A significant increase in ERS markers GRP78, ATF4, and CHOP was observed in CDCA-stimulated HepG2 cells, and SSD intervention suppressed this trend (Fig. 4D). CDCA-stimulated HepG2 cells were incubated with ERS agonist TM or inhibitor TUDCA (Fig. 4E). TUDCA, similar to SSD intervention, was shown to reverse the decrease in cell viability and increase in cytotoxicity and apoptosis rate caused by CDCA (Fig. 4F-H). Whereas TM could counteract the protective effect of SSD intervention on CDCA-stimulated HepG2 cells (Fig. 4F-H).

#### SSD inhibits TLR4/MyD88/NF-κB/NLRP3 pathway activation

**Fig. 5:**
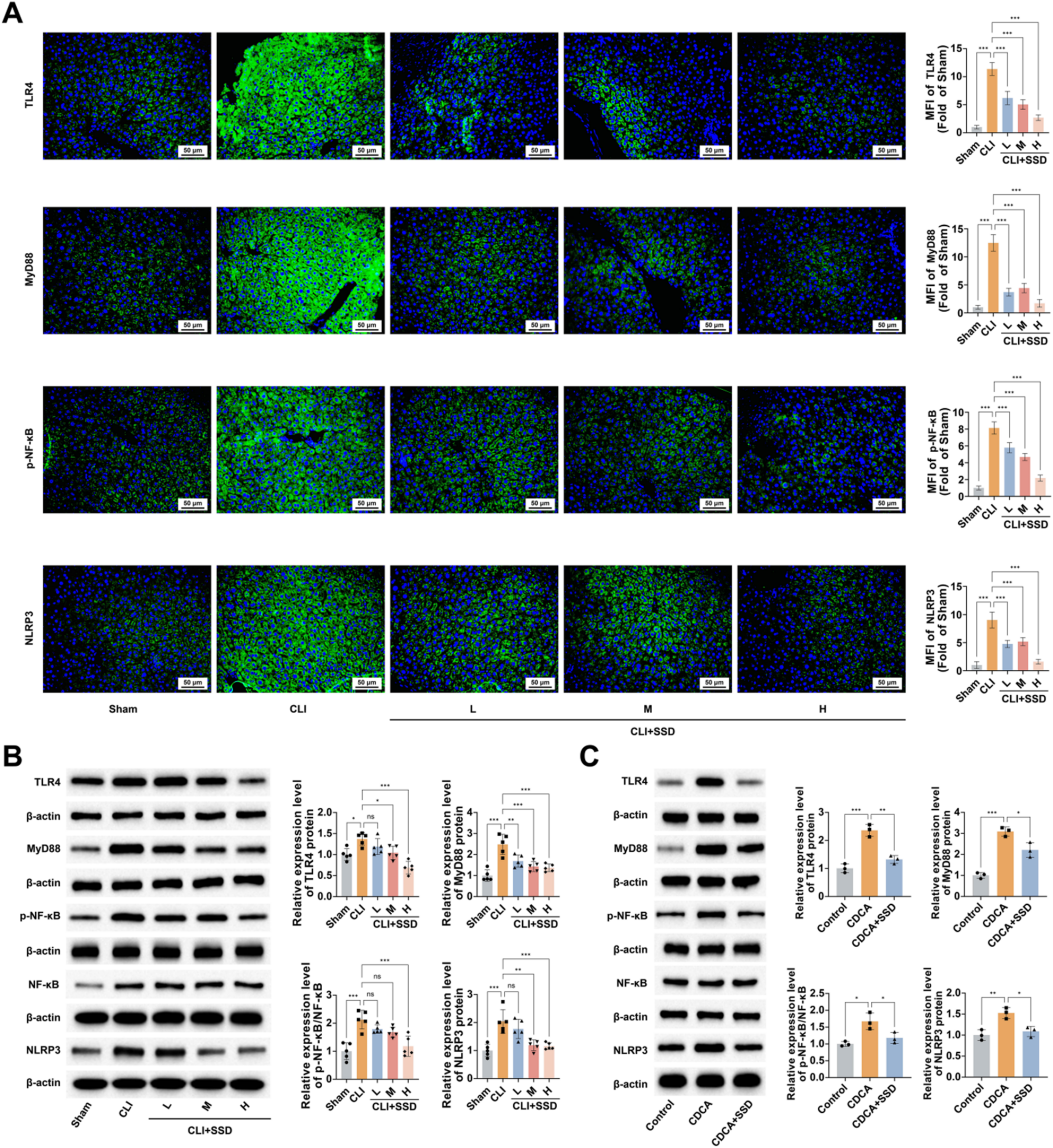
SSD inhibits TLR4/MyD88/NF-κB/NLRP3 pathway activation. **A**: Immunofluorescence to detect the effect of SSD on hepatic TLR4/MyD88/NF-κB/NLRP3 pathway protein expression in CLI mice, and the corresponding Mean Fluorescence Intensity (MFI) quantitative analysis (*n* = 5); **B**: Western blot to detect the effect of SSD on hepatic TLR4/MyD88/NF-κB/NLRP3 pathway protein expression in CLI mice (*n* = 5); **C**: Western blot to detect the effect of SSD on CDCA-stimulated TLR4/MyD88/NF-κB/NLRP3 pathway protein expression in HepG2 cells (*n* = 3). Data are presented as mean ± SD. * *P* < 0.05, ** *P* < 0.01, *** *P* < 0.001, ns, not significant.

BDL significantly induced elevated protein expression and promoted TLR4/MyD88/NF-κB/NLRP3 pathway activation in mouse liver by immunofluorescence and Western blot detection (Fig. 5A, B). SSD inhibited BDL-induced TLR4/MyD88/NF-κB/NLRP3 pathway activation. CDCA induced TLR4/MyD88/NF-κB/NLRP3 pathway activation in HepG2 cells, which was inhibited by SSD intervention (Fig. 5C).

### SSD inhibits bile acid-induced ERS in HepG2 cells via the TLR4/MyD88/NF-κB/NLRP3 pathway

**Fig. 6:**
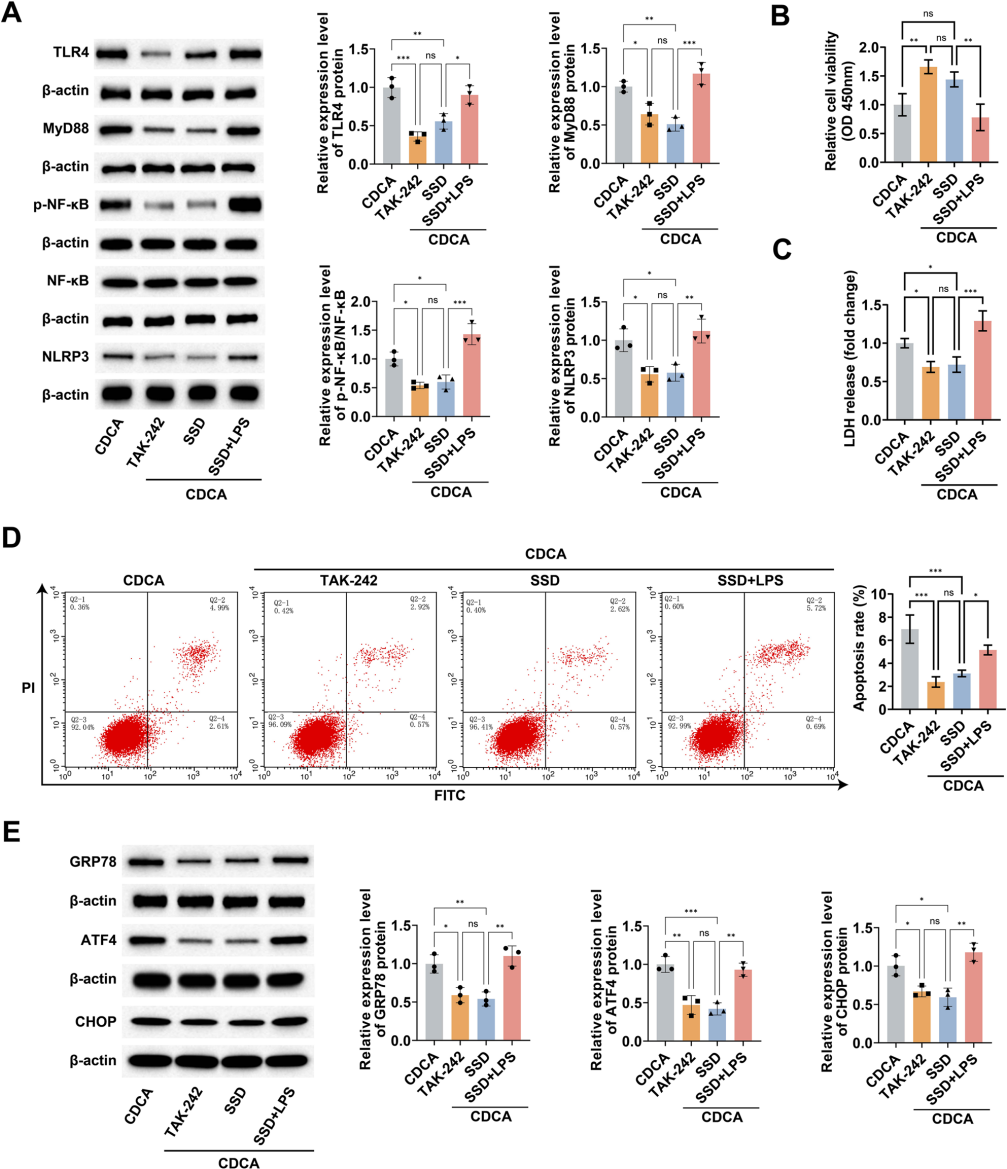
SSD inhibits bile acid-induced ERS in HepG2 cells via TLR4/MyD88/NF-κB/NLRP3 pathway. **A**: Western blot to detect the effect of LPS or TAK-242 on the protein expression of TLR4/MyD88/NF-κB/NLRP3 pathway in CDCA-stimulated HepG2 cells; **B**: CCK8 to detect the effect of LPS or TAK-242 on the viability of CDCA-stimulated HepG2 cells; **C**: LDH release to assess the effect of LPS or TAK-242 on the toxicity of CDCA-stimulated HepG2 cells; **D**: Flow cytometry to detect the effect of LPS or TAK-242 on the apoptosis rate of CDCA-stimulated HepG2 cells; **E**: Western blot to detect the effect of LPS or TAK-242 on the protein expression of ATF4, CHOP and GRP78 in CDCA-stimulated HepG2 cells. Data are presented as mean ± SD (*n* = 3). * *P* < 0.05, ** *P* < 0.01, *** *P* < 0.001, ns, not significant

CDCA-stimulated HepG2 cells were treated with TLR4 agonist LPS or TLR4 inhibitor TAK-242 (Fig. 6A). TAK-242 and SSD interventions had similar effects, reversing CDCA-induced decrease in cell viability and elevated cytotoxicity, and reducing CDCA-induced elevated apoptosis rate (Fig. 6B-D). More importantly TAK-242 likewise inhibited CDCA-induced elevated expression of ERS markers GRP78, ATF4 and CHOP (Fig. 6E). In opposition, LPS nullified the protective effect of SSD intervention on CDCA-stimulated HepG2 cells and its inhibitory action on ERS (Fig. 6B-E).

## Discussion

CLI is characterized by metabolic disorders and excessive accumulation of intrahepatic bile acids [24]. Bile acid buildup, oxidative stress, inflammation, and programmed cell death are key factors in CLI pathogenesis, contributing to disease progression and representing potential targets for therapy [10]. BDL is a well-established mouse model for the study of cholestatic liver disease, which mimics the biliary obstruction and hepatic and serum bile acid accumulation observed in human patients [25]. This study observed that SSD treatment was effective in ameliorating BDL-induced hepatic impairment, bile acid accumulation, hepatocyte necrosis, collagen deposition, and hepatocyte apoptosis. In the cholestatic state, bile acids accumulate in the liver, causing ERS, oxidative stress, and mitochondrial damage in hepatocytes, while bile acids-injured hepatocytes release high levels of inflammatory cytokines [26]. SSD has a protective effect against liver injury by inhibiting inflammatory responses and antioxidants [21]. Consistent with this, our results also showed that SSD treatment effectively reduced BDL-induced hepatic inflammation and oxidative stress.

Under normal conditions, three transmembrane proteins (IRE1α, PERK, and ATF6) in the endoplasmic reticulum interact with immunoglobulin heavy chain binding protein (Bip)/GRP78, in an inactive state [27]. ERS triggers the dissociation of transmembrane proteins from GRP78, allowing it to bind to unfolded or misfolded proteins, and the elevation of GRP78/Bip is recognized as a marker of ERS activation [28]. When the ERS pathway is activated, PREK undergoes phosphorylation, which activates its downstream molecules. It specifically binds to eIF2α, thereby rendering it incapable of translating proteins and inhibiting protein translation. When ERS is not restored, eIF2α promotes the translation of ATF4 mRNA, which in turn upregulates CHOP at the transcriptional level [29]. CHOP is a key regulator of multiple modes of ERS-induced hepatocyte death including autophagy, apoptosis, and pyroptosis [30]. Our results showed that GRP78, ATF4, and CHOP were significantly upregulated in BDL livers, suggesting that BDL induced hepatic ERS. Whereas GRP78, ATF4, and CHOP proteins decreased after SSD intervention. SSD treatment reduces protein expression of ERS-related proteins p-eIF2α, ATF4, and CHOP in NAFLD mice [21]. CDCAs are the most toxic and potent bile acids, a trait determined by their hydrophobic nature. It has been demonstrated that CDCAs promote hepatocyte ERS, ROS generation, and inflammation, ultimately leading to apoptosis [31]. In this research, SSD effectively alleviated CDCA-induced damage and ERS in HepG2 cells. And ERS agonist TM could counteract the protective effect of SSD intervention on CDCA-stimulated HepG2 cells, and SSD may ameliorate CDCA-induced HepG2 cell injury by inhibiting ERS. Therefore, it is hypothesized that the anti-inflammatory, antioxidant and hepatoprotective effects of SSD in CLI may be related to the modulation of ERS. The interaction mechanism or pathway between ERS and oxidative stress or inflammation in the context of SSD modulation may need additional study.

SSD inhibits pilocarpine-induced elevation of TLR4 expression in astrocytes and suppresses NLRP3/caspase-1 [32]. In contrast, in LPS-induced acute lung injury, SSD significantly reduces the elevated NF-κB/TLR4 pathway-related protein expression [33]. SSD can attenuate hepatic fibrosis by negatively regulating ROS/NLRP3 inflammasome [14]. Our study found that SSD blocked the BDL-induced increase in hepatic TLR4/MyD88/NF-κB/NLRP3 pathway protein expression in vivo and the CDCA-stimulated rise in this pathway’s protein expression in HepG2 cells in vitro. Furthermore, TLR4 inhibitor TAK-242, similar to SSD, ameliorated CDCA-induced HepG2 cell injury and ERS. TLR4 agonist LPS, on the other hand, counteracted the protective effect of SSD intervention on CDCA-stimulated HepG2 cells. It has been noted that ERS induction in HepG2 cells could be blocked by TAK-242 [34]. In contrast, HMGB1 can trigger activation of the PERK-ATF4-CHOP signaling pathway to induce ERS in a TLR4-dependent manner [35]. Our results therefore suggest that SSD ultimately attenuated BDL-induced CLI by inhibiting ERS. Mechanistically, these effects may be related to the regulation of the TLR4/MyD88/NF-κB/NLRP3 pathway. However, the interactions between the TLR4 pathway and ERS are complex, and the molecular mechanisms involved between the TLR4/MyD88/NF-κB/NLRP3 pathway and the ERS signaling pathway during SSD regulation need to be further investigated in the future.

## Conclusion

**Fig. 7:**
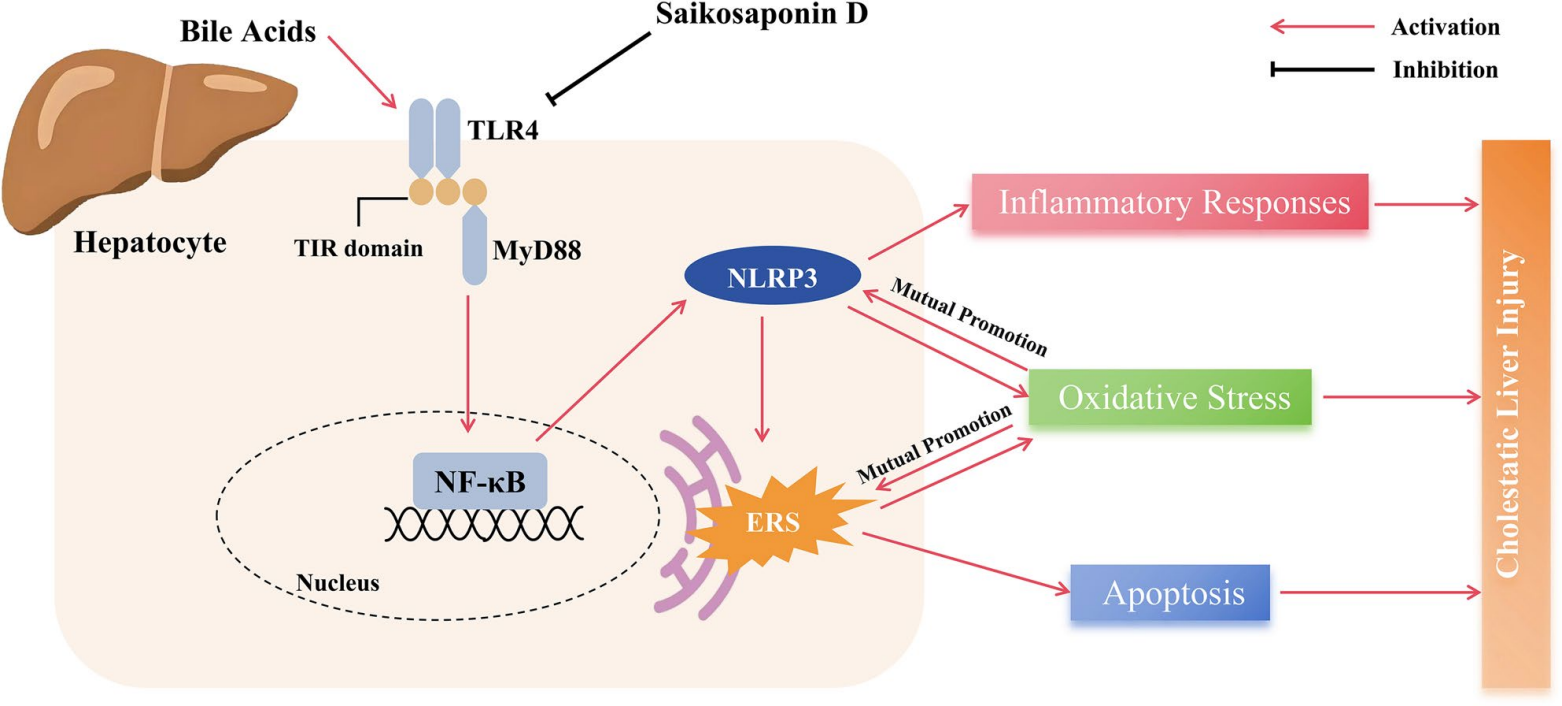
Schematic illustrating the protective effect and mechanism of SSD treatment in cholestatic liver injury. SSD attenuates oxidative stress, inflammation, apoptosis, and endoplasmic reticulum stress through inhibition of the TLR4/MyD88/NF-κB/NLRP3 axis

In conclusion, SSD alleviates BDL-induced CLI. The underlying mechanism involves, at least in part, the TLR4/MyD88/NF-κB/NLRP3 pathway, a signaling pathway that may be associated with ERS (Fig. 7). These data suggest that SSD is an effective drug for the treatment of CLI.

## Supplementary Information

Supplementary Material 1.

## Data Availability

Data is available from the corresponding author on request.

## References

[1] Frissen M, Liao L, Schneider KM, Djudjaj S, Haybaeck J, Wree A, et al. Bidirectional Role of NLRP3 During Acute and Chronic Cholestatic Liver Injury. Hepatology. 2021;73(5):1836–54. 32748971 10.1002/hep.31494

[2] Li J, Zhu X, Zhang M, Zhang Y, Ye S, Leng Y, et al. Limb expression 1-like (LIX1L) protein promotes cholestatic liver injury by regulating bile acid metabolism. J Hepatol. 2021;75(2):400–13. 33746084 10.1016/j.jhep.2021.02.035

[3] Wang Y, Fu X, Zeng L, Hu Y, Gao R, Xian S, et al. Activation of Nrf2/HO-1 signaling pathway exacerbates cholestatic liver injury. Commun Biol. 2024;7(1):621. 38783088 10.1038/s42003-024-06243-0 PMC11116386

[4] Wagner M, Fickert P. Drug Therapies for Chronic Cholestatic Liver Diseases. Annu Rev Pharmacol Toxicol. 2020;60:503–27. 31506007 10.1146/annurev-pharmtox-010818-021059

[5] Mazzetti M, Marconi G, Mancinelli M, Benedetti A, Marzioni M, Maroni L. The Management of Cholestatic Liver Diseases: Current Therapies and Emerging New Possibilities. J Clin Med. 2021;10(8):1763. 10.3390/jcm10081763 PMC8073106 33919600

[6] Oakes SA, Papa FR. The role of endoplasmic reticulum stress in human pathology. Annu Rev Pathol. 2015;10:173–94. 25387057 10.1146/annurev-pathol-012513-104649 PMC5568783

[7] Marciniak SJ, Chambers JE, Ron D. Pharmacological targeting of endoplasmic reticulum stress in disease. Nat Rev Drug Discov. 2022;21(2):115–40. 34702991 10.1038/s41573-021-00320-3

[8] Hetz C, Zhang K, Kaufman RJ. Mechanisms, regulation and functions of the unfolded protein response. Nat Rev Mol Cell Biol. 2020;21(8):421–38. 32457508 10.1038/s41580-020-0250-z PMC8867924

[9] Liu X, Green RM. Endoplasmic reticulum stress and liver diseases. Liver Res. 2019;3(1):55–64. 32670671 10.1016/j.livres.2019.01.002 PMC7363397

[10] Pan PH, Wang YY, Lin SY, Liao SL, Chen YF, Huang WC et al. 18β-Glycyrrhetinic Acid Protects against Cholestatic Liver Injury in Bile Duct-Ligated Rats. Antioxid (Basel). 2022;11(5):961. 10.3390/antiox11050961 PMC9138139 35624826

[11] Mosaoa RM, Al-Rabia MW, Asfour HZ, Alhakamy NA, Mansouri RA, El-Agamy DS, et al. Targeting SIRT1/AMPK/Nrf2/NF-кB by sitagliptin protects against oxidative stress-mediated ER stress and inflammation during ANIT-induced cholestatic liver injury. Toxicology. 2024;507:153889. 10.1016/j.tox.2024.153889 39029735

[12] Gu S, Zheng Y, Chen C, Liu J, Wang Y, Wang J, Li Y. Research progress on the molecular mechanisms of Saikosaponin D in various diseases (Review). Int J Mol Med. 2025;55(3):37. 10.3892/ijmm.2024.5478 PMC11722148 39717942

[13] Chen Y, Que R, Lin L, Shen Y, Liu J, Li Y. Inhibition of oxidative stress and NLRP3 inflammasome by Saikosaponin-d alleviates acute liver injury in carbon tetrachloride-induced hepatitis in mice. Int J Immunopathol Pharmacol. 2020;34:2058738420950593. 32816567 10.1177/2058738420950593 PMC7444099

[14] Zhang K, Lin L, Zhu Y, Zhang N, Zhou M, Li Y. Saikosaponin d Alleviates Liver Fibrosis by Negatively Regulating the ROS/NLRP3 Inflammasome Through Activating the ERβ Pathway. Front Pharmacol. 2022;13:894981. 35694250 10.3389/fphar.2022.894981 PMC9174603

[15] Chen SN, Tan Y, Xiao XC, Li Q, Wu Q, Peng YY, et al. Deletion of TLR4 attenuates lipopolysaccharide-induced acute liver injury by inhibiting inflammation and apoptosis. Acta Pharmacol Sin. 2021;42(10):1610–9. 33495514 10.1038/s41401-020-00597-x PMC8463538

[16] Zhang SH, Yu MJ, Yan JL, Xiao JH, Xiao Y, Yang JL, et al. TLR4 Knockout Attenuates BDL-induced Liver Cholestatic Injury through Amino Acid and Choline Metabolic Pathways. Curr Med Sci. 2021;41(3):572–80. 34047945 10.1007/s11596-021-2364-8

[17] Su J, Pan YW, Wang SQ, Li XZ, Huang F, Ma SP. Saikosaponin-d attenuated lipopolysaccharide-induced depressive-like behaviors via inhibiting microglia activation and neuroinflammation. Int Immunopharmacol. 2020;80:106181. 31926446 10.1016/j.intimp.2019.106181

[18] Wang X, Li S, Yu J, Wang W, Du Z, Gao S, et al. Saikosaponin B2 ameliorates depression-induced microglia activation by inhibiting ferroptosis-mediated neuroinflammation and ER stress. J Ethnopharmacol. 2023;316:116729. 37277081 10.1016/j.jep.2023.116729

[19] Liu XL, Fan L, Yue BH, Lou Z. Saikosaponin A mitigates the progression of Parkinson’s disease via attenuating microglial neuroinflammation through TLR4/MyD88/NF-κB pathway. Eur Rev Med Pharmacol Sci. 2023;27(15):6956–71. 37606106 10.26355/eurrev_202308_33268

[20] Bai Y, Li K, Li X, Chen X, Zheng J, Wu F, et al. Effects of oxidative stress on hepatic encephalopathy pathogenesis in mice. Nat Commun. 2023;14(1):4456. 37488119 10.1038/s41467-023-40081-8 PMC10366183

[21] Chang GR, Lin WL, Lin TC, Liao HJ, Lu YW. The Ameliorative Effects of Saikosaponin in Thioacetamide-Induced Liver Injury and Non-Alcoholic Fatty Liver Disease in Mice. Int J Mol Sci. 2021;22(21):11383. 10.3390/ijms222111383 PMC8583725 34768813

[22] Han D, Kim H, Kim S, Le QA, Han SY, Bae J, et al. Sestrin2 protects against cholestatic liver injury by inhibiting endoplasmic reticulum stress and NLRP3 inflammasome-mediated pyroptosis. Exp Mol Med. 2022;54(3):239–51. 35260799 10.1038/s12276-022-00737-9 PMC8980001

[23] Pan PH, Wang YY, Lin SY, Liao SL, Chen YF, Huang WC, et al. Plumbagin ameliorates bile duct ligation-induced cholestatic liver injury in rats. Biomed Pharmacother. 2022;151:113133. 35594710 10.1016/j.biopha.2022.113133

[24] Hu Z, Cheng X, Cai J, Huang C, Hu J, Liu J. Emodin alleviates cholestatic liver injury by modulating Sirt1/Fxr signaling pathways. Sci Rep. 2024;14(1):16756. 39033253 10.1038/s41598-024-67882-1 PMC11271454

[25] Zhuang Y, Ortega-Ribera M, Thevkar Nagesh P, Joshi R, Huang H, Wang Y, et al. Bile acid-induced IRF3 phosphorylation mediates cell death, inflammatory responses, and fibrosis in cholestasis-induced liver and kidney injury via regulation of ZBP1. Hepatology. 2024;79(4):752–67. 37725754 10.1097/HEP.0000000000000611 PMC10948324

[26] Cai SY, Boyer JL. The role of bile acids in cholestatic liver injury. Ann Transl Med. 2021;9(8):737. 33987435 10.21037/atm-20-5110 PMC8106037

[27] Schwarz DS, Blower MD. The endoplasmic reticulum: structure, function and response to cellular signaling. Cell Mol Life Sci. 2016;73(1):79–94. 26433683 10.1007/s00018-015-2052-6 PMC4700099

[28] Wang M, Kaufman RJ. Protein misfolding in the endoplasmic reticulum as a conduit to human disease. Nature. 2016;529(7586):326–35. 26791723 10.1038/nature17041

[29] Zielke S, Kardo S, Zein L, Mari M, Covarrubias-Pinto A, Kinzler MN, et al. ATF4 links ER stress with reticulophagy in glioblastoma cells. Autophagy. 2021;17(9):2432–48. 33111629 10.1080/15548627.2020.1827780 PMC8496713

[30] Zhang J, Guo J, Yang N, Huang Y, Hu T, Rao C. Endoplasmic reticulum stress-mediated cell death in liver injury. Cell Death Dis. 2022;13(12):1051. 36535923 10.1038/s41419-022-05444-x PMC9763476

[31] Lee D, Park JS, Kim D, Hong HS. Substance P Hinders Bile Acid-Induced Hepatocellular Injury by Modulating Oxidative Stress and Inflammation. Antioxid (Basel). 2022;11(5):920. 10.3390/antiox11050920 PMC9137937 35624784

[32] Liu JY, Shen YL, Zhu JY, Yang DD. Saikosaponin D mitigate pilocarpine-induced astrocyte injury by regulating the NLRP3/caspase-1 signaling pathway. Chem Biol Drug Des. 2024;103(3):e14481. 38458969 10.1111/cbdd.14481

[33] Peng D, Chen Y, Sun Y, Zhang Z, Cui N, Zhang W et al. Saikosaponin A and Its Epimers Alleviate LPS-Induced Acute Lung Injury in Mice. Molecules. 2023;28(3):967. 10.3390/molecules28030967 PMC9919285 36770631

[34] Wang T, Wang M, Liu W, Zhang L, Zhang J, Zhao J et al. Intracellular CIRP promotes liver regeneration via STAT3 signaling pathway activation after partial hepatectomy in mice. Int J Mol Med. 2025;55(3):42. 10.3892/ijmm.2025.5483 PMC11758893 39791211

[35] Wang R, Feng W, Wang Y, Jiang Y, Lin Y, Chen X. Maternal obstructive sleep apnea aggravates metabolic dysfunction-associated fatty liver disease via HMGB1-TLR4 signaling-mediated endoplasmic reticulum stress in male offspring rats. Biochim Biophys Acta Mol Basis Dis. 2024;1870(1):166889. 37730152 10.1016/j.bbadis.2023.166889

