# Supplementary material for: Protective effect of Saikosaponin D modulating endoplasmic reticulum stress mediated by TLR4/MyD88/NF-κB/NLRP3 pathway on cholestatic liver injury
Source: Hereditas. 2026 Apr 11;163:62. doi: 10.1186/s41065-026-00669-8 (PMC13181951; doi:10.1186/s41065-026-00669-8)
Supplement: Supplementary file 1 — Supplementary Material 1. [file 41065_2026_669_MOESM1_ESM.pdf]

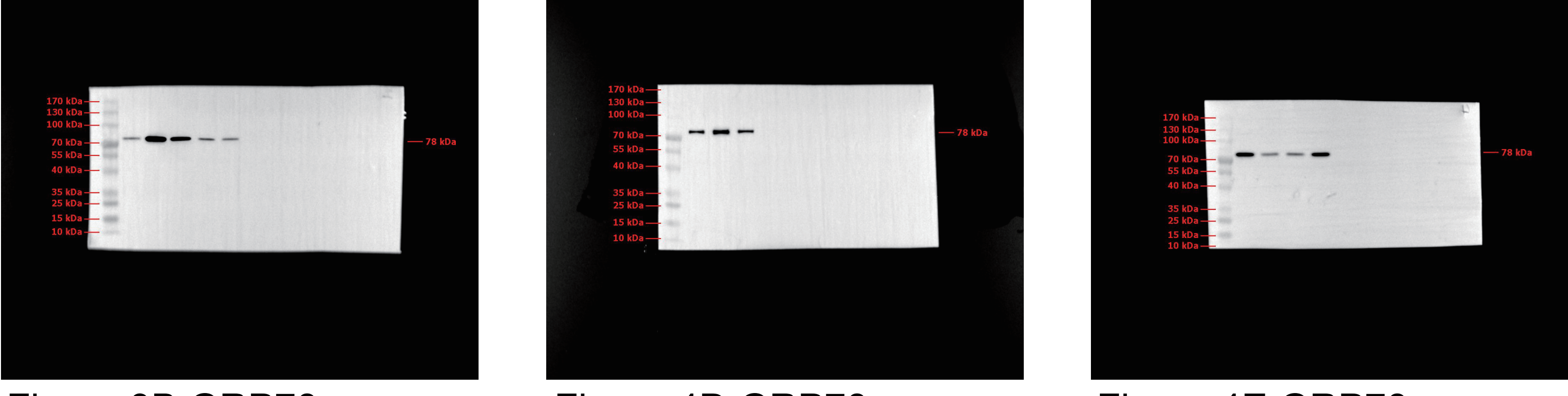

Figure 3B GRP78 | Figure 4D GRP78 | Figure 4E GRP78

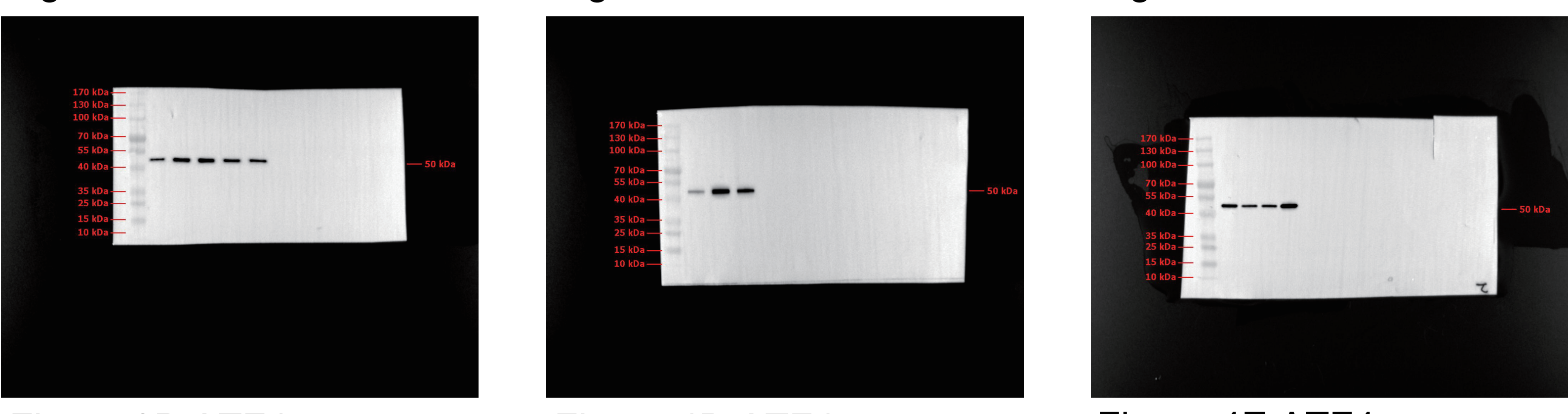

Figure 3B ATF4 | Figure 4D ATF4 | Figure 4E ATF4

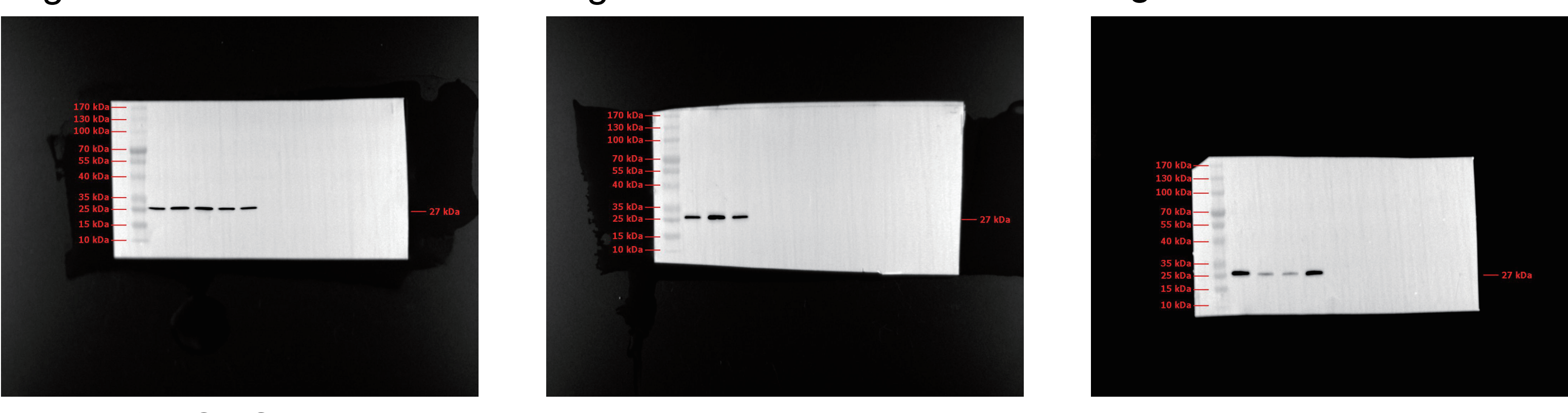

Figure 3B CHOP | Figure 4D CHOP | Figure 4E CHOP

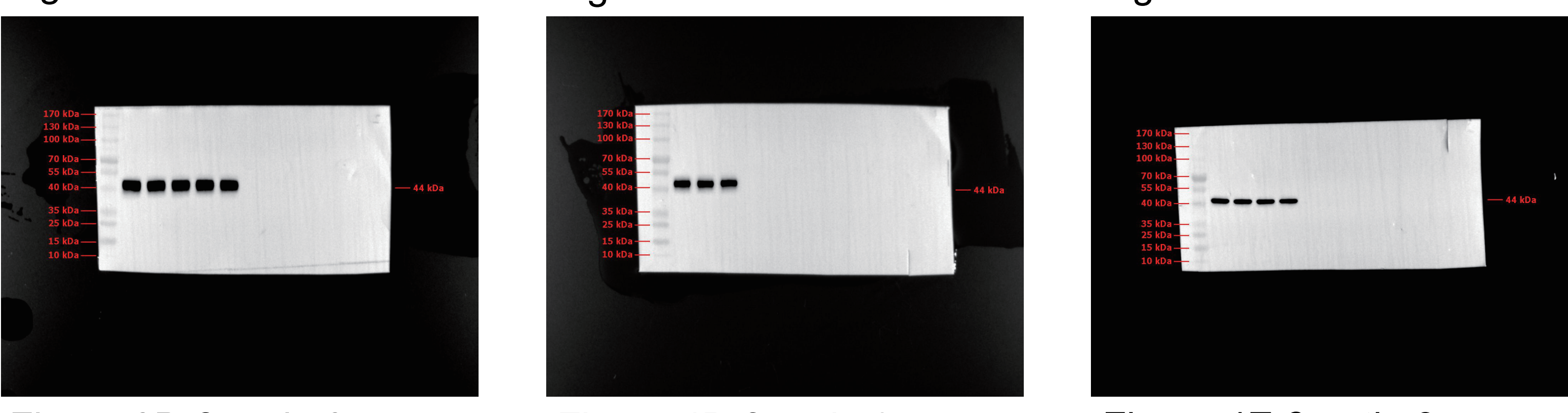

Figure 3B β-actin 3 | Figure 4D β-actin 3 | Figure 4E β-actin 3

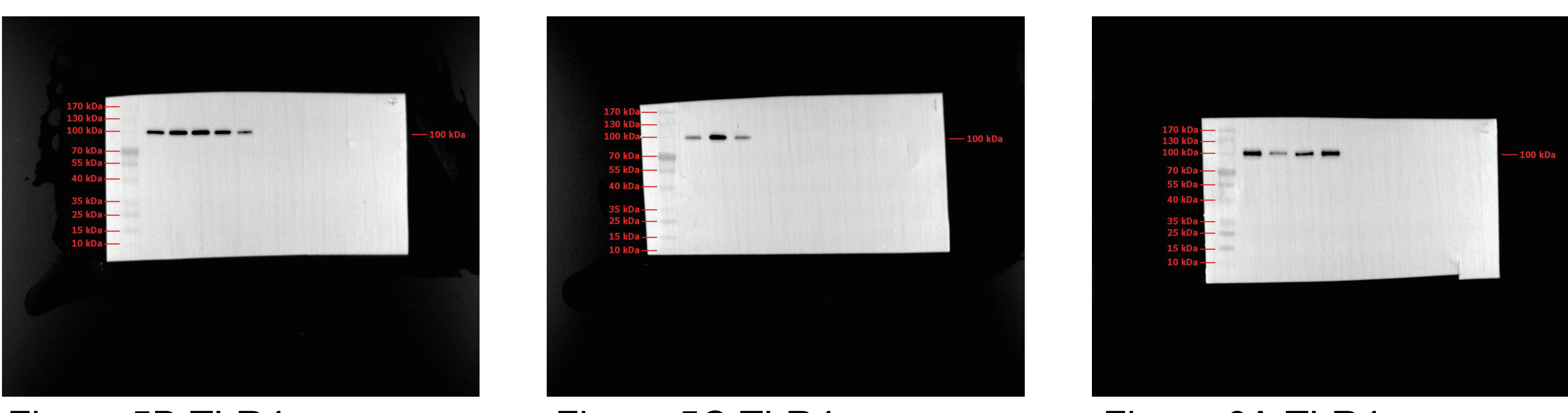

Figure 5B TLR4 | Figure 5C TLR4 | Figure 6A TLR4

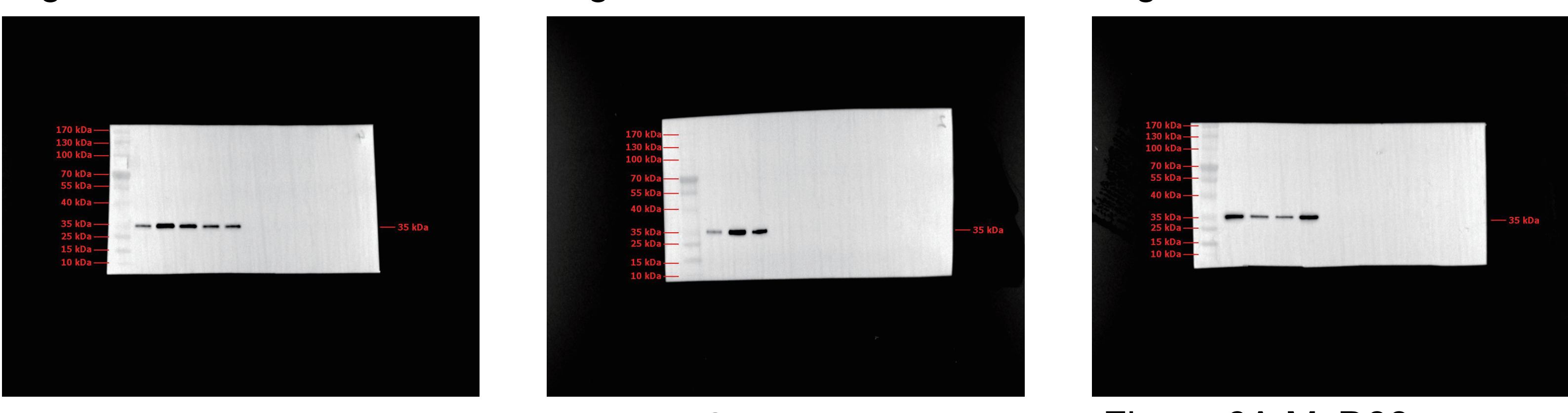

Figure 5B MyD88 | Figure 5C MyD88 | Figure 6A MyD88

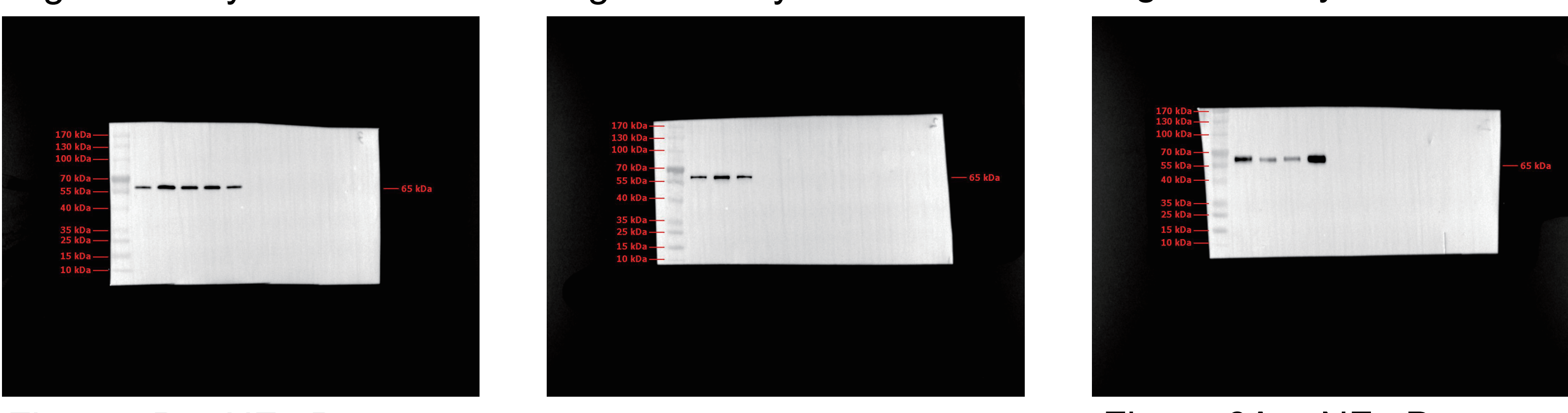

Figure 5B p-NF-κB | Figure 5C p-NF-κB | Figure 6A p-NF-κB

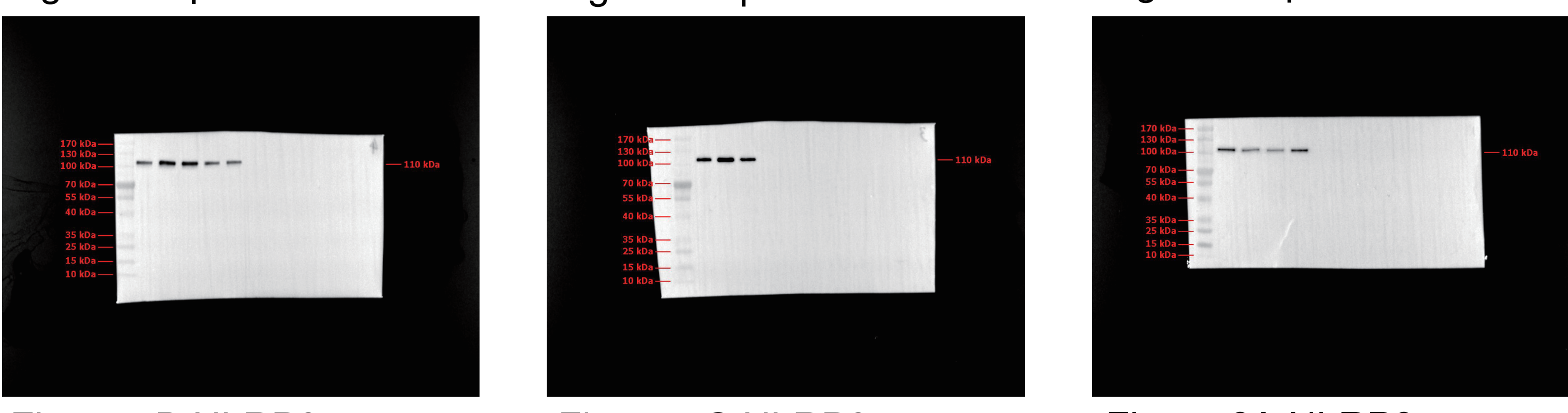

Figure 5B NLRP3 | Figure 5C NLRP3 | Figure 6A NLRP3

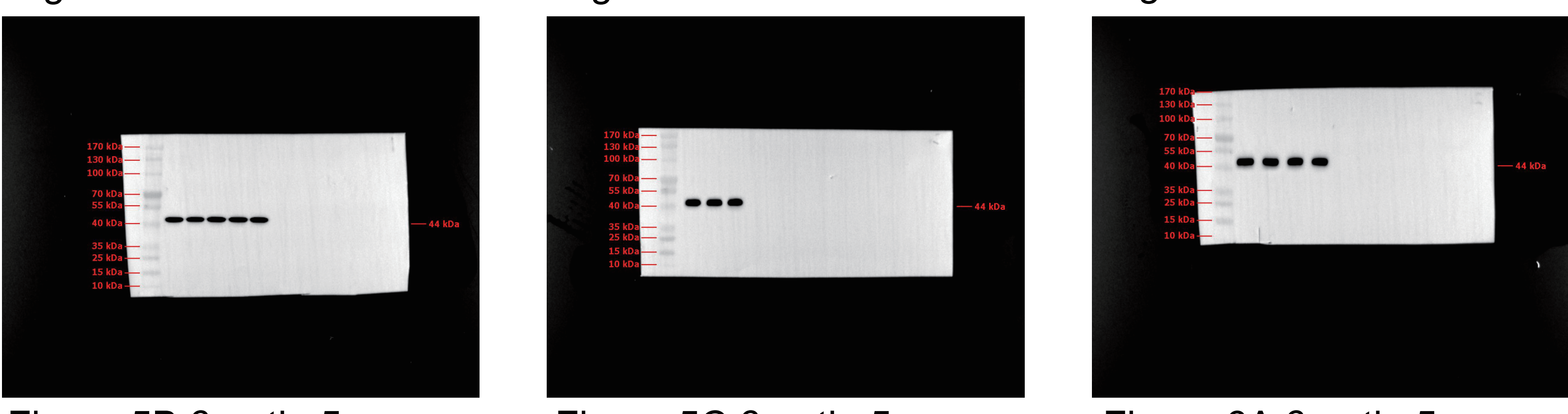

Figure 5B β-actin 5 | Figure 5C β-actin 5 | Figure 6A β-actin 5

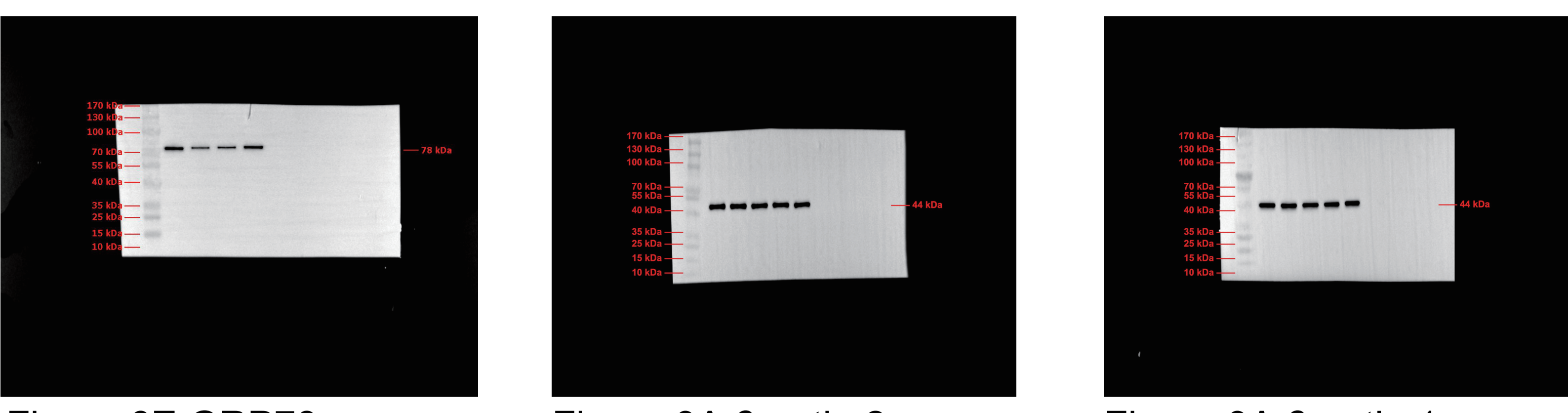

Figure 6E GRP78 | Figure 3A β-actin 2 | Figure 3A β-actin 1

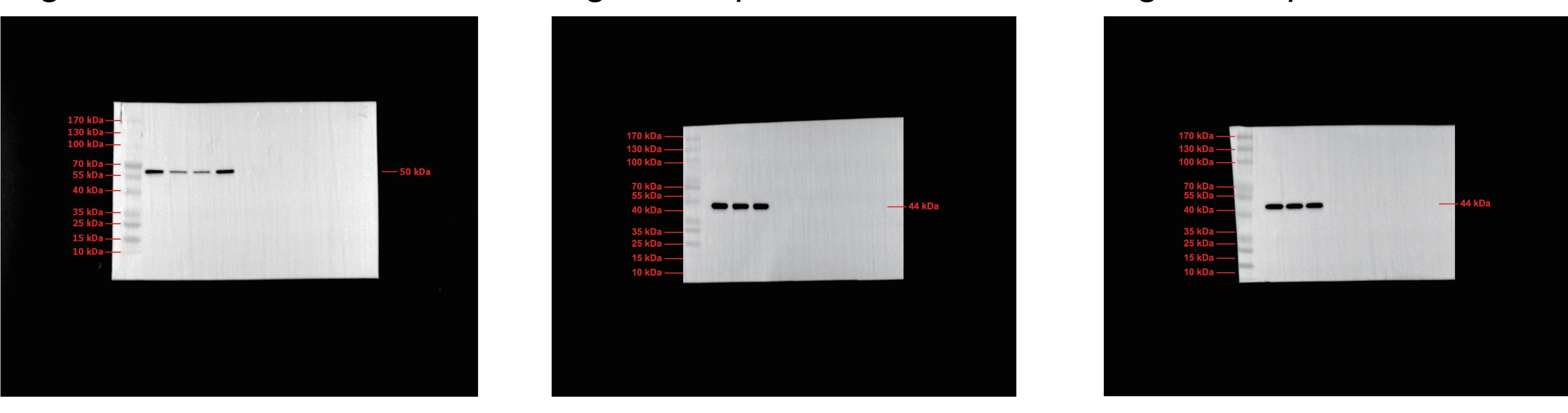

Figure 6E ATF4 | Figure 4D β-actin 1 | Figure 4D β-actin 2

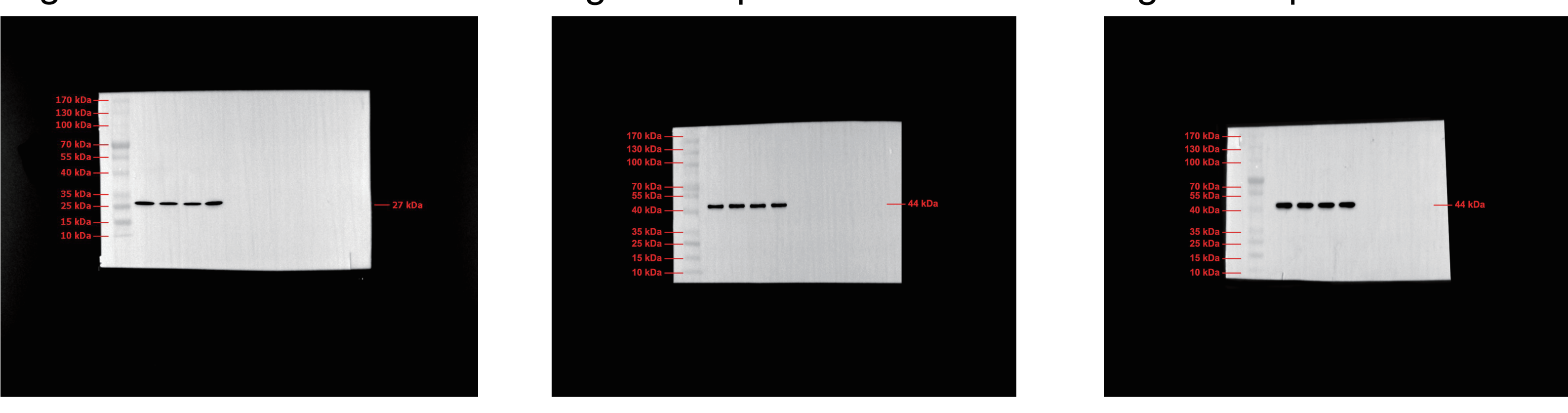

Figure 6E CHOP | Figure 4E β-actin 1 | Figure 4E β-actin 2

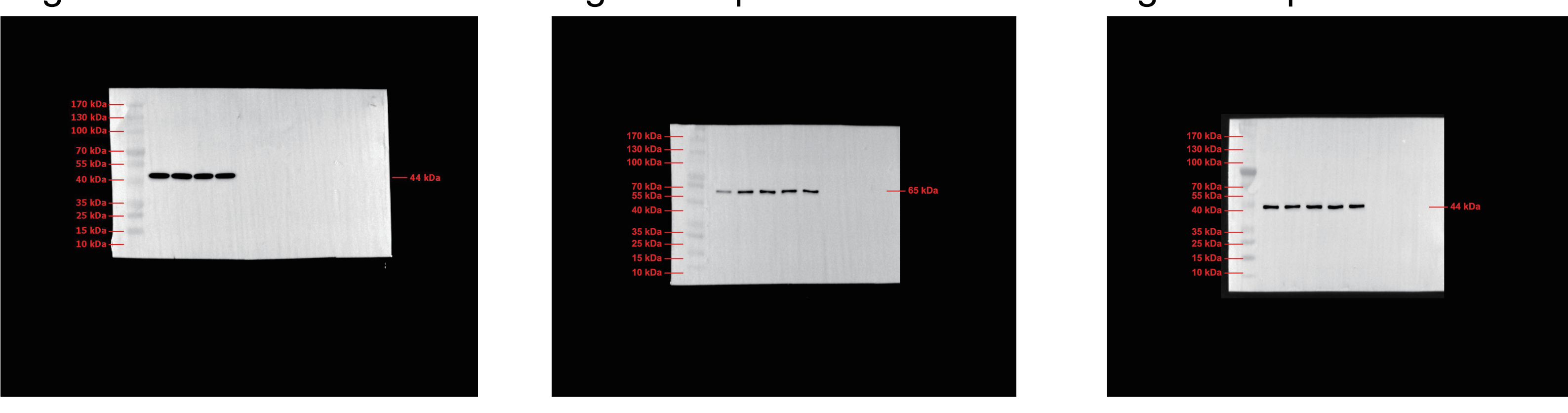

Figure 6E β-actin 3 | Figure 5B NF-κB | Figure 5B β-actin 1

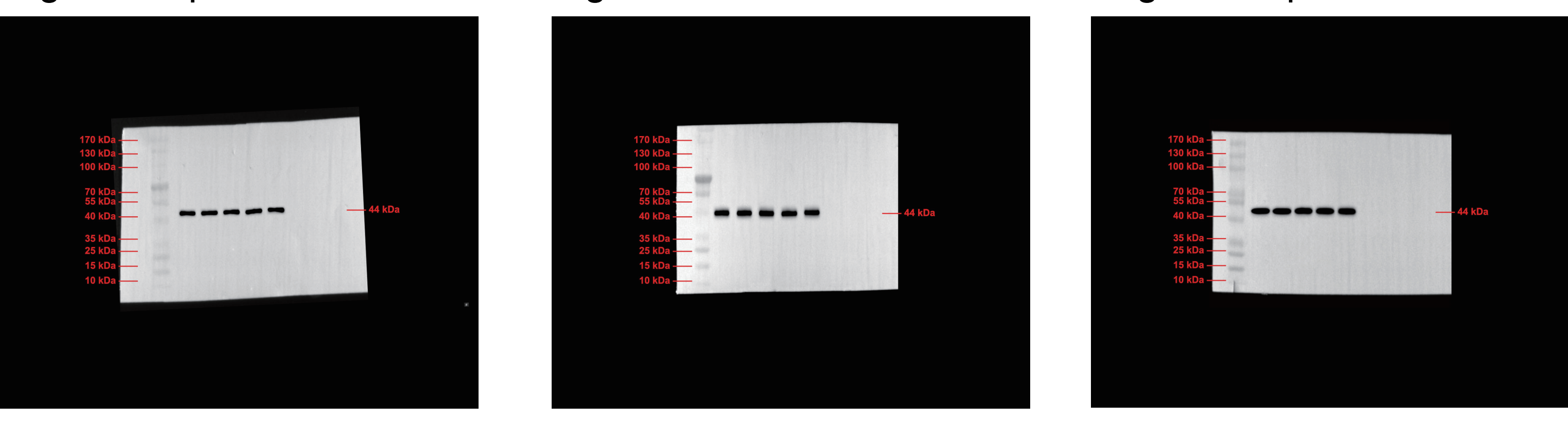

Figure 5B β-actin 2 | Figure 5B β-actin 3 | Figure 5B β-actin 4

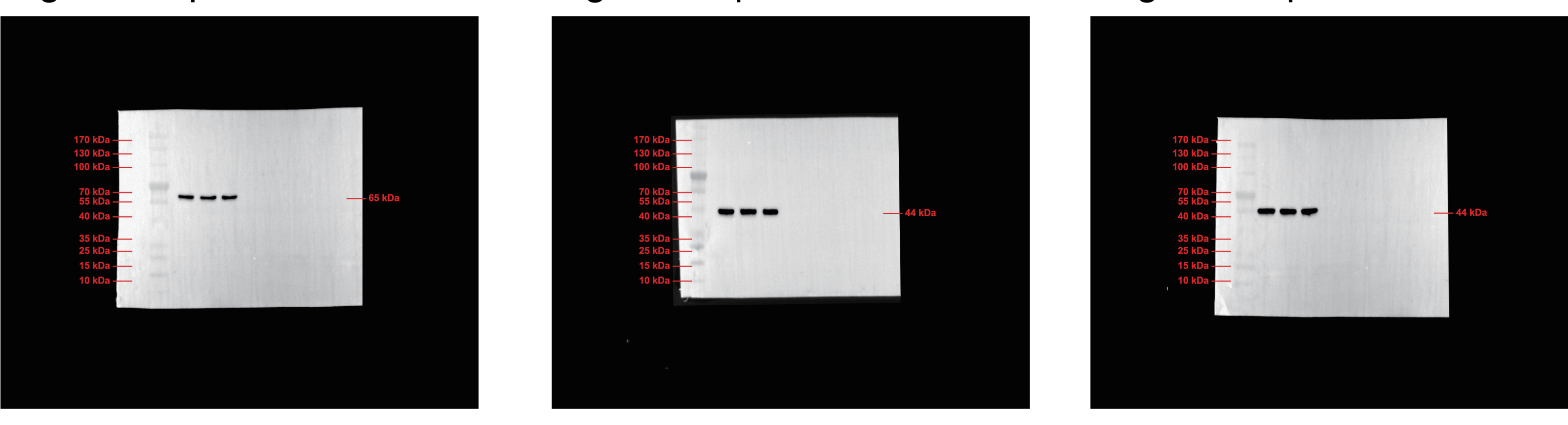

Figure 5C NF-κB | Figure 5C β-actin 1 | Figure 5C β-actin 2

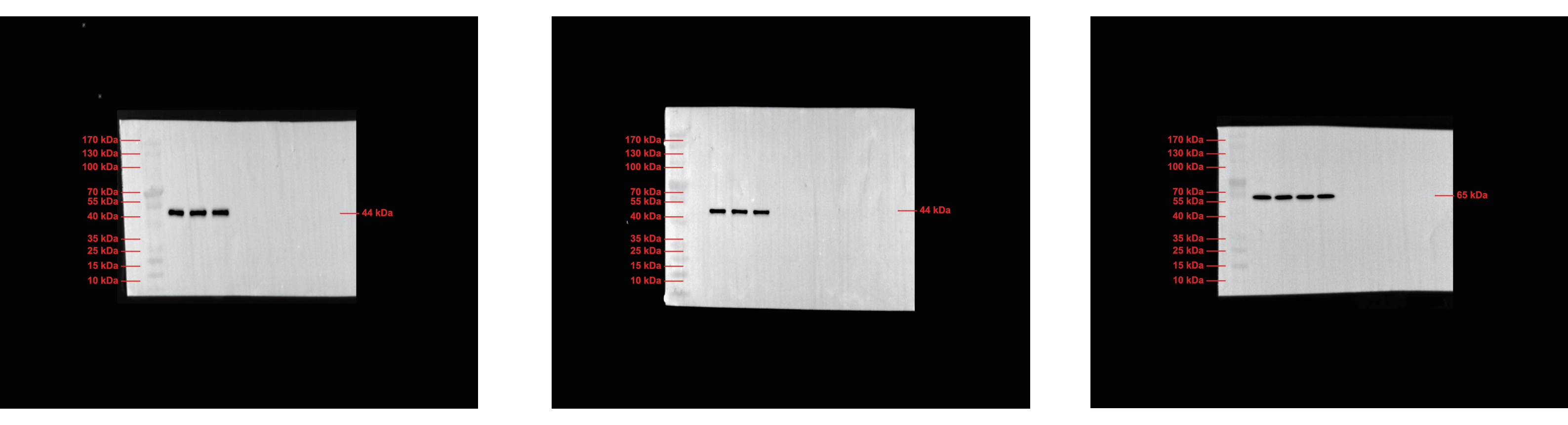

Figure 5C β-actin 3 | Figure 5C β-actin 4 | Figure 6A NF-κB

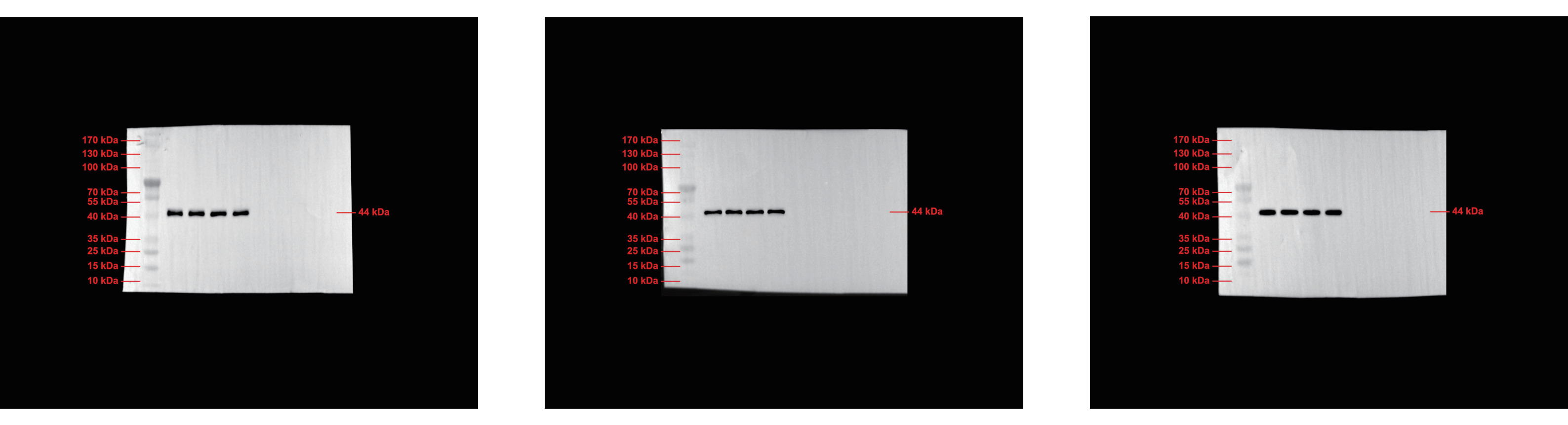

Figure 6A β-actin 1 | Figure 6A β-actin 2 | Figure 6A β-actin 3

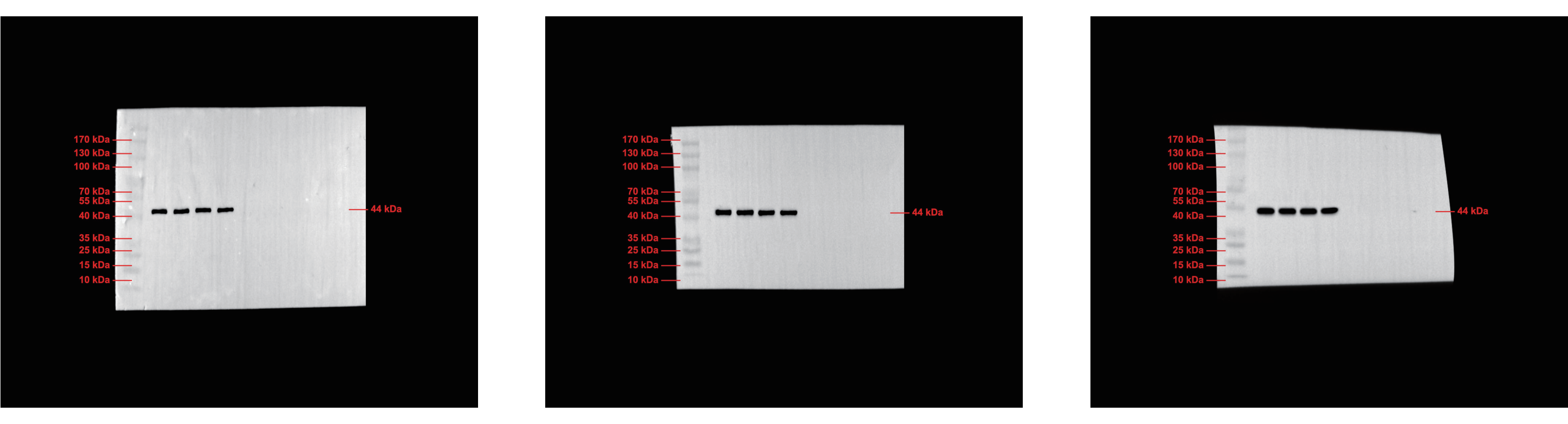

Figure 6A β-actin 4 | Figure 6E β-actin 1 | Figure 6E β-actin 2
